# Standards of treatment in Forensic Mental Health: A Systematic Review

**DOI:** 10.1192/j.eurpsy.2023.922

**Published:** 2023-07-19

**Authors:** J. Uden, B. Völlm, D. Cerci

**Affiliations:** Clinic for Forensic Psychiatry, Rostock University Medical Center (UMR), Rostock, Germany

## Abstract

**Introduction:**

In Forensic Mental Health, standards of treatment offer a legal, ethical and organizational backbone for professionals facing challenging patients and complex procedures. Grounded in UN resolutions, standards implement human rights and ethical principles in forensic psychiatry. Guidelines establish recommendations for optimizing patient care and agreements on minimum standards. Internationally, diverse approaches to standards and guidelines have developed due to differing medicolegal systems.

**Objectives:**

This review´s objective was to provide insight into which areas are considered essential in standards of treatment and guidelines in forensic psychiatry. Furthermore, we aimed to investigate if American Psychological Association (APA) principles for the publication and implementation of guidelines were applied and if European Committee for the Prevention of Torture and Inhuman or Degrading Treatment or Punishment (CPT) criteria were considered.

**Methods:**

A systematic literature search was carried out. PubMed, Psyindex, Livivo, Scopus, Google Search and Google Scholar were searched for records published by August 2022. The following search terms were used in different variations and combinations: “forensic", “mental health”, “psychiatry”, "standards”, “treatment” “service provision “principles” “quality” “indicators” “Forensische Psychiatrie” “Maßregelvollzug” and “Qualitätsindikatoren”. Standards, guidelines and reviews in Forensic Mental Health in English and German were included. The guidelines were assessed by applying APA principles for guidelines and CPT recommendations.

**Results:**

The search identified 12 documents. Eight documents were excluded as they were focusing only on models of care, forensic evaluation or were in the state of a discussion paper for one specific healthcare system. Four publications from Australia, Canada, Germany and UK were included in narrative synthesis. The selected documents vary in scope, objective, thematic focus on ethical or practical aspects, and level of detail. Our assessment showed that APA-recommended elements of a guideline were often missing. The guidelines discussed were also not fully compliant with CPT recommendations. A more extensive source citation is often needed. In total, “Standards for Forensic Mental Health Services” (UK, 2021) demonstrated good compliance with APA and CPT criteria and comparatively the best practical applicability.

**Conclusions:**

This systematic review indicates that standards and guidelines in forensic mental health still require improvement in terms of formal frameworks of medical guidelines. Human rights compliance in forensic psychiatry must be continued to be monitored and standards of treatment and guidelines offer an important opportunity to ensure adherence. Further research on the implementation of standards into day-to-day procedures is needed.

**Disclosure of Interest:**

None Declared

